# Best friends’ and adolescents’ depressive symptoms in early adolescence: gendered pathways and distinct roles of peer acceptance

**DOI:** 10.3389/fpsyg.2026.1909249

**Published:** 2026-08-19

**Authors:** Yiqun Yang, Xiangjuan Tian, Qiuxu Zhang

**Affiliations:** 1 School of Marxism, Shandong University of Technology, Zibo, China; 2 School of Psychology, Shandong Second Medical University, Weifang, China

**Keywords:** adolescents’ depressive symptoms, best friends’ depressive symptoms, friendship quality, gender, peer acceptance, rumination

## Abstract

**Introduction:**

Although best friends’ depressive symptoms have been linked to adolescents’ own depressive symptoms, inconsistent findings point to the need to examine potential pathways and the peer-status contexts in which these links unfold.

**Methods:**

Using cross-sectional data from 939 Chinese early adolescents (Mage = 13.31 years; 49.2% girls), this study tested whether best friends’ depressive symptoms were associated with adolescents’ depressive symptoms through friendship quality and rumination, and whether these associations varied according to peer acceptance of best friends and adolescents themselves. Depressive symptoms, friendship quality, and rumination were assessed using self-reports, whereas peer acceptance and best-friend identification were obtained through peer nominations.

**Results:**

Among girls, best friends’ depressive symptoms were indirectly associated with adolescents’ depressive symptoms through poorer friendship quality and greater rumination. Among boys, these indirect pathways were not significant; however, the association between best friends’ depressive symptoms and boys’ own symptoms was stronger when best friends’ peer acceptance was higher. Among girls, higher peer acceptance was related to a weaker association between rumination and depressive symptoms and a smaller indirect association through rumination.

**Discussion:**

These findings highlight potentially different patterns across gender-stratified analyses and the peer-status contexts of both adolescents and their best friends in understanding adolescent depressive symptoms.

## 1 Introduction

Adolescence is a period of heightened vulnerability to depression, during which peer relationships become increasingly central to youths’ emotional and social adjustment (Schwartz-Mette et al., 2020). Best friends can promote positive adjustment through social support and protection, yet they may also facilitate the sharing or contagion of maladaptive outcomes (Rudolph et al., 2016). Although a growing body of Western research has documented an association between adolescents’ depressive symptoms and those of their friends (e.g., Giletta et al., 2011; Prinstein, 2007), findings have been inconsistent. Thus, examining potential pathways that may help account for this link is necessary to advance our understanding of this association. The present study examines whether best friends’ depressive symptoms are associated with adolescents’ own depressive symptoms among Chinese boys and girls. Informed by relational and cognitive perspectives (Coyne, 1976; Nolen-Hoeksema, 1991), we further investigate friendship quality and rumination as two theory-based potential pathways, and explore whether adolescents’ own peer acceptance and best friends’ peer acceptance moderate the corresponding associations.

### 1.1 Depressive symptom similarity among adolescent best friends

Depressive symptoms among adolescent best friends are likely to be linked because close friendships provide a salient context for emotional sharing and interpersonal adaptation (Rudolph et al., 2016). Homophily and peer influence perspectives suggest that adolescents may affiliate with emotionally similar peers and may also become more similar to their friends through repeated interaction and mutual adjustment (Brechwald and Prinstein, 2011). During adolescence, friends become increasingly important sources of emotional support and self-definition, and adolescents may attend to, share, and adapt to friends’ emotional expressions and coping responses (Rudolph et al., 2016). Consistent with social learning and identity-based perspectives (Abrams and Hogg, 1990; Bandura, 1986), and with the influence-compatibility model (Laursen and Veenstra, 2021), depressive symptom similarity may be understood as part of broader interpersonal processes through which adolescents maintain closeness and reduce dissimilarity within valued friendships.

Prior research has shown that adolescents’ depressive symptoms are associated with those of their best friends, but the evidence remains mixed. Two meta-analytic reviews suggest that peer influence effects on depressive symptoms are generally significant but small (Giletta et al., 2021), and that only 4 of 7 studies found evidence that adolescents’ depressive symptoms became more similar to their peers’ depressive symptoms over time (Neal and Veenstra, 2021). Some longitudinal studies have documented a direct positive association, suggesting that depressive symptoms may be transmitted between close friends (Giletta et al., 2011; Stevens and Prinstein, 2005). However, other research indicates that this association is not uniform, but may depend on gender, friendship closeness, peer status, social anxiety, and the broader relational context. For example, Giletta et al. (2012) found that depressive symptom similarity with very best friends over time occurred only among girls. Similarly, Prinstein (2007) reported that susceptibility to friends’ depressive symptoms varied by individual characteristics: girls with higher social anxiety were more vulnerable, whereas boys were more susceptible when their friends were perceived as popular by peers. In contrast, a recent study by Bernasco et al. (2023) found no evidence that close friends’ depressive symptoms predicted adolescents’ later depressive symptoms. Taken together, existing research suggests that depressive symptom similarity among best friends is better understood as a context-dependent phenomenon, shaped by relationship quality, individual vulnerabilities, gender, and broader peer-group status. This provides the basis for the present study’s focus on examining when and for whom this association may be more evident.

### 1.2 Interpersonal and cognitive pathways: friendship quality and rumination

The association between best friends’ depressive symptoms and adolescents’ own depressive symptoms may be explained by two pathways: an interpersonal pathway through friendship quality and a cognitive pathway through rumination. The interpersonal theory of depression proposes that depressed individuals may engage in maladaptive interpersonal behaviors such as excessive reassurance seeking and negative feedback seeking—that eventually induce negative affect in their close others and reduce the quality of close relationships (Conway et al., 2011; Coyne, 1976). Poorer friendship quality may then reduce emotional support and belongingness, both of which are closely related to adolescents’ depressive symptoms (Li et al., 2025; Yang et al., 2020). Consistent with this view, depressive symptoms have been found to interfere with adolescents’ development of adaptive social skills and to elicit negative responses from peers and friends (Agoston and Rudolph, 2013; Kochel et al., 2012). Considering the establishment of close, stable, and intimate friendships as important tasks for adolescent maturation (Waters and Sroufe, 1983), difficulties in maintaining high-quality friendships may increase vulnerability to depressive symptoms (Allen et al., 2024). A meta-analytic review of 233 studies found small but significant concurrent and longitudinal associations between youths’ friendship experiences and depressive symptoms, indicating that friendship functioning is meaningfully linked to emotional adjustment in adolescence (Schwartz-Mette et al., 2020). These findings support the possibility that friendship quality may serve as a potential interpersonal pathway in the association between best friends’ depressive symptoms and adolescents’ own depressive symptoms.

The cognitive pathway focuses on the role of rumination as a negative thinking style. Complementing the interpersonal perspective, Response Styles Theory (RST) defines rumination as a relatively stable cognitive response to depressed mood, characterized by a passive and repetitive focus on symptoms of distress and the causes and consequences of that distress (Nolen-Hoeksema, 1991). Although no study has directly examined the role of rumination in the link between best friends’ depressive symptoms and adolescents’ own depressive symptoms, indirect evidence supports this possibility. First, when adolescents interact with friends who report higher depressive symptoms, they may become involved in an interpersonal form of rumination—co-rumination—which may reinforce individual ruminative tendencies (Aldrich et al., 2019; Spendelow et al., 2017). Indeed, studies have found that co-rumination predicts increases in adolescents’ rumination, which in turn heightens their depressive symptoms (Bastin et al., 2021; Stone and Gibb, 2015). Second, substantial empirical evidence indicates that rumination is associated with greater depressive symptoms among adolescents (e.g., Hankin, 2008). Based on this evidence, it is reasonable to hypothesize that best friends’ depressive symptoms may be indirectly associated with adolescents’ depressive symptoms through greater rumination.

### 1.3 Distinct roles of peer acceptance

Although depressive symptoms may be linked within close friendships, such an association is likely shaped by both adolescents’ own characteristics and the characteristics of their best friend (Brechwald and Prinstein, 2011). As a broader social context, peer status is an important feature of adolescents’ interpersonal interactions, as youth and their friends may occupy different positions within the peer group (La Greca and Harrison, 2005). Peer acceptance, as a key indicator of peer status, is associated with adolescents’ depressive symptoms and may work together with best-friendship characteristics in relation to adolescents’ adjustment (Kochel et al., 2012; Prinstein, 2007). Thus, the association between best friends’ depressive symptoms and adolescents’ own depressive symptoms may be influenced by the peer acceptance of both members of the friendship dyad. Importantly, best friends’ peer acceptance and adolescents’ own peer acceptance may have distinct implications within this process. Best friends’ peer acceptance represents a partner-level characteristic that reflects the friend’s relational standing and potential social salience within the peer group, whereas adolescents’ own peer acceptance reflects the broader relational resources available to the adolescent (Brechwald and Prinstein, 2011; Birkeland et al., 2014; Kochel et al., 2017). Thus, the former may shape the social salience of the friendship partner’s characteristics, whereas the latter may shape the resources available to the recipient of that influence.

Existing evidence suggests that socially valued peers may be particularly influential. Peer influence models suggest that adolescents are more likely to attend to, imitate, and internalize the behaviors and emotional styles of socially salient peers (Brechwald and Prinstein, 2011). High-status peers may serve as a “reference group” shaping social norms, to which other adolescents are more likely to conform in their behaviors and emotional styles (Choukas-Bradley et al., 2015). Thus, when a best friend is highly accepted, their emotional expressions, coping style, and interpretations of stress may carry greater interpersonal salience within the friendship and broader peer context (Slagter et al., 2023). Prinstein’s (2007) work found that higher levels of friends’ peer-perceived popularity were associated with greater susceptibility to depressive symptom contagion in boys. A highly accepted best friend may receive greater attention and social validation, making the friend’s emotional expressions, coping responses, and interpretations of stressful experiences more salient to the adolescent (Brechwald and Prinstein, 2011; Choukas-Bradley et al., 2015; Slagter et al., 2023). Best friends’ peer acceptance may therefore increase the extent to which the friend’s depressive characteristics become socially consequential within the friendship (Brechwald and Prinstein, 2011), although existing evidence does not establish whether this influence is expressed primarily through friendship functioning, cognitive processing, or adolescents’ depressive symptoms more directly.

Adolescents’ own peer acceptance, in contrast, may shape their access to resources that protect against the emotional consequences of interpersonal and cognitive vulnerability (Birkeland et al., 2014; Kochel et al., 2017). Adolescents who are broadly accepted may have access to alternative sources of support, more opportunities for positive peer interaction, and a stronger sense of belonging (Birkeland et al., 2014). These resources may buffer the emotional costs of having a best friend with higher depressive symptoms. Conversely, adolescents with low peer acceptance may be especially vulnerable because the best friendship may become a primary or even exclusive interpersonal context (Waldrip et al., 2008), increasing susceptibility to the friends’ depressive symptoms and to maladaptive interpersonal and cognitive processes. For example, Conway et al. (2011) found that the depression socialization effect was most potent among adolescents on the periphery of peer groups. Other research indicates that high levels of peer acceptance protect adolescents from risks associated with depressive symptoms (Kochel et al., 2017). Broader peer acceptance may not necessarily prevent difficulties within a particular friendship or eliminate adolescents’ tendency to ruminate; instead, the support, belonging, and positive interactions associated with peer acceptance may reduce the extent to which these interpersonal and cognitive vulnerabilities are associated with adolescents’ own depressive symptoms.

This distinction also helps explain why peer relationships in adolescence should not be treated as a single undifferentiated domain. Although best friendships and peer-group status may be linked, they organize adolescents’ social lives at different levels and may operate through different mechanisms (Rudolph et al., 2016). Accordingly, best friends’ peer acceptance may reflect the friendship partner’s relational standing and social salience, whereas adolescents’ own peer acceptance may reflect access to protective resources within the wider peer ecology.

### 1.4 Gender as a contextual factor

Gender differences in adolescents’ responses to interpersonal experiences have been widely documented, particularly in the domain of depressive symptoms (Poulin and Chan, 2010; Rose and Rudolph, 2006). Several frameworks highlight gendered patterns in social–emotional development. Gender socialization and relational-cultural theories propose that girls are socialized to value intimacy, emotional expression, and relational interdependence, whereas boys are often encouraged toward autonomy and emotional restraint (Brody and Hall, 2010; Rose and Rudolph, 2006). Consequently, girls’ sense of self and emotional well-being may be more strongly tied to dyadic friendships. Similarly, the cognitive-interpersonal model further suggests that girls are more likely to engage in maladaptive cognitive and interpersonal coping strategies, such as rumination, which can amplify emotional distress (Hankin et al., 2010). Research on depressive symptom contagion indicates that adolescents’ depressive symptoms can become more similar to those of their friends over time, with some evidence suggesting stronger or more consistent effects in girls’ friendships (Prinstein, 2007; Stevens and Prinstein, 2005). At the same time, it is important to note that findings are not entirely uniform, and boys are not immune to interpersonal influences. Some studies suggest that under certain conditions—such as high levels of peer stress—boys may also exhibit significant susceptibility to peer processes (Rose and Rudolph, 2006). Thus, rather than assuming categorical differences, gender may function as a contextual factor that shapes the strength or form of interpersonal processes. Given variability in prior findings and the complexity of these processes, the present study adopts a gender-stratified approach, examining the proposed associations separately among boys and girls rather than specifying directional hypotheses.

### 1.5 The present study

Building on previous research on depressive symptom similarity between best friends, the present study adopts a more integrative approach to examining the interpersonal, cognitive, and peer-contextual factors associated with this link. By simultaneously incorporating friendship quality, rumination, adolescents’ own peer acceptance, best friends’ peer acceptance, and gender within a unified moderated mediation framework, this study extends existing research beyond the direct association between friends’ depressive symptoms and examines how this association is embedded in multiple interpersonal and peer contexts.

**Figure 1:**
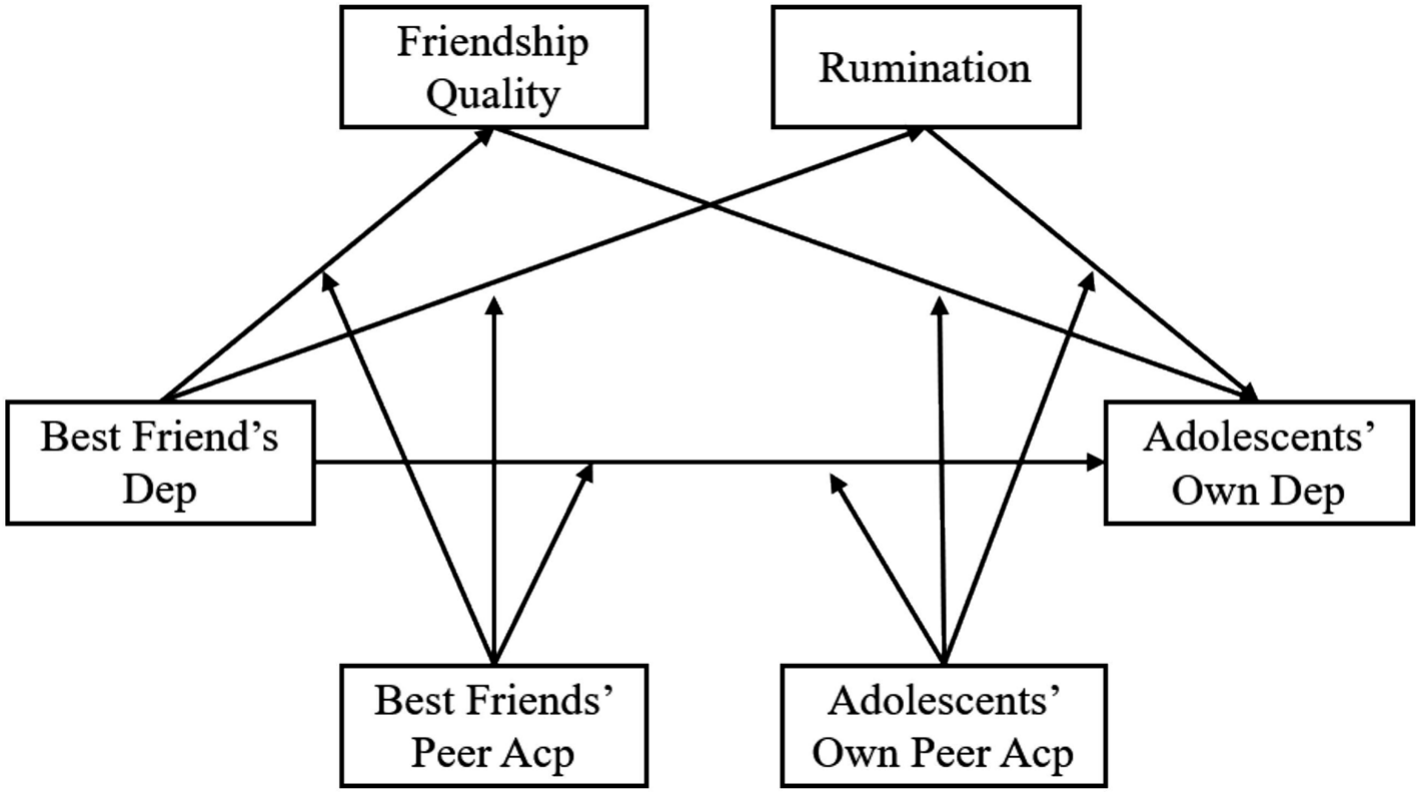
Conceptual model of the association between best friends’ and adolescents’ depressive symptoms. Dep = depressive symptoms, Acp = acceptance.

Accordingly, the present study aimed to examine how adolescents’ depressive symptoms are embedded in both dyadic friendship processes and broader peer-group status. Guided by interpersonal models of adolescent depression and peer influence perspectives, we focus on best friends’ depressive symptoms as a key interpersonal correlate of adolescents’ depressive symptoms. As shown in the hypothesized model (see Figure 1), best friends’ depressive symptoms are expected to be associated with adolescents’ depressive symptoms both directly and indirectly through friendship quality and rumination. Friendship quality may capture the interpersonal context involved in the association between best friends’ depressive symptoms and adolescents’ emotional adjustment, whereas rumination may represent a cognitive-emotional process potentially involved in the association between best friends’ and adolescents’ depressive symptoms.

This study further distinguishes between two forms of peer acceptance that may serve different functions within the proposed model. Best friends’ peer acceptance was conceptualized as a partner-level indicator of the friend’s social visibility and potential influence. We therefore expected higher best-friend peer acceptance to increase the potential influence associated with best friends’ depressive symptoms. In contrast, adolescents’ own peer acceptance was conceptualized as an indicator of the broader relational resources available to adolescents and was therefore expected to serve as a protective context. However, because previous research has provided limited evidence regarding the specific pathways through which these two forms of peer acceptance operate, we did not formulate pathway-specific moderation hypotheses. The moderation of the individual component paths was examined in a theoretically informed but exploratory manner. In addition, the proposed model was examined separately among boys and girls, given prior evidence of gender-related variation in sensitivity to interpersonal influences, although no specific directional hypotheses are proposed. Overall, this study tests an integrated moderated mediation model to clarify when, for whom, and through which interpersonal and cognitive processes best friends’ depressive symptoms are linked to adolescents’ depressive symptoms.

## 2 Method

### 2.1 Participants and procedures

The initial sample consisted of 1,024 adolescents (524 boys, *M*_age_ = 13.31 years, *SD* = 1.03) who participated in a school-based research project, the Chinese Children and Adolescents Development Project (CCADP). Data for this study were collected from two middle schools (22 classes, Grades 6–8) located in Shandong Province, China. Of the initial sample, 85 adolescents did not provide a best-friend nomination. Because friendship quality was assessed with reference to the nominated friend, and the friend’s depressive symptoms and peer acceptance were obtained by linking the nomination to that classmate’s data, these variables were unavailable for these participants. They were therefore excluded from the analytic sample, resulting in a final sample of 939 adolescents. Participants without nomination data had significantly lower levels of peer acceptance than those with nomination data, although the magnitude of this difference was modest (|Cohen’s *d*| = 0.46). Specifically, participants with missing nomination data scored relatively lower in adolescents’ own peer acceptance than those without missing nomination data.

The current study was approved by the Ethics Committee of the Shandong University of Technology. Informed assent from adolescents and consent from parents and school principals were obtained prior to data collection. During a single class period at schools (approximately 40 min), adolescents completed a battery of self-report measures regarding their depressive symptoms and social experiences and peer nominations. Each participant received a gift worth approximately $2.

### 2.2 Measures

#### 2.2.1 Depressive symptoms

Depressive symptoms were assessed with the Chinese version of the Center for Epidemiologic Studies Depression Scale (CES-D, Radloff, 1977), a 20-item self-report measure assessing depressive symptoms experienced in the past week on a 4-point scale from 0 (rarely) to 3 (most or all of the time). Scores were averaged, with higher scores indicating greater depressive symptom severity. The Chinese version of the CES-D has demonstrated good construct validity in Chinese children and adolescents (Zhu et al., 2021). Cronbach’s alpha was 0.84 in this study.

#### 2.2.2 Friendship quality

The quality of friendship was assessed using the Chinese revised version of the Friendship Quality Questionnaire (FQQ; Parker and Asher, 1993). Participants first nominated one best friend from their classroom and then completed the items with reference to this nominated best friend. This adapted version consists of 18 items and has been validated in Chinese samples (Zhou et al., 2023). Each item is rated on a 5-point Likert scale (1 = not at all true to 5 = really true), with higher scores indicating higher perceived friendship quality. Cronbach’s alpha was 0.83 in this study.

#### 2.2.3 Rumination

Rumination was assessed using the Ruminative Responses Scale (RRS; Nolen-Hoeksema and Morrow, 1991), a 22-item self-report measure on which participants rate how frequently they engage in ruminative thoughts when feeling sad or depressed (1 = almost never to 4 = almost always). Higher average scores reflect a greater tendency toward rumination. Cronbach’s alpha was 0.91 in this study.

#### 2.2.4 Adolescents’ own peer acceptance

A standardized peer-nomination procedure was used to collect information on adolescents’ own peer acceptance (Coie et al., 1982). Students were asked to nominate any classmates whom they liked most, without a limit on the number of nominations. The nominations received by each student were summed and then standardized within each class to permit appropriate comparisons. Higher scores represented higher levels of peer acceptance.

#### 2.2.5 Best friend’s depressive symptoms and peer acceptance

A peer-nomination procedure was used to identify adolescents’ best friends (Parker and Asher, 1993). Adolescents were asked to select one “best friend” from a roster of all classmates alphabetized by first name. For each adolescent, the nominated best friend’s CES-D and liked-most nomination score were used as indicators of best friends’ depressive symptoms and peer acceptance.

### 2.3 Analytical strategy

Statistical analyses were conducted using SPSS 26.0 and Mplus 8.3. First, descriptive statistics and correlations among the key study variables were examined, and Harman’s single-factor test and a bifactor model were used to assess potential common method bias (Podsakoff et al., 2012). Second, measurement invariance across gender was tested using Mplus 8.3. A series of increasingly restrictive confirmatory factor analysis (CFA) models were specified to examine configural, metric, scalar, and strict invariance across boys and girls (Vandenberg and Lance, 2000). Then, the mediating effects and the moderated mediation analyses were tested using the PROCESS macro v5.0 (Hayes, 2022) with 5,000 bias-corrected bootstrap samples. Effects were considered significant when the 95% confidence interval (CI) did not include zero. All mediation and moderated mediation analyses were conducted separately for boys and girls. Specifically, PROCESS Model 4 was used to test the parallel mediating effects of friendship quality and rumination in the association between best friends’ depressive symptoms and adolescents’ depressive symptoms. PROCESS Models 8 and 15 were used to probe the moderating effects of best friends’ peer acceptance and adolescents’ own peer acceptance on the corresponding associations in the mediation model.

All analyses controlled for grade and only-child status. Grade was included to account for developmental and school-context differences across Grades 6–8, as associations between depressive symptoms and peer relationships may vary across developmental stages (Yang et al., 2020). Grade was used instead of age because the two substantially overlap, whereas grade more directly reflects the classroom-based context in which peer nominations were obtained. Only-child status was included as a contextually relevant family-structure characteristic associated with psychological adjustment among Chinese adolescents (Falbo and Hooper, 2015; Zhang et al., 2024).

Gender-stratified analyses were conducted because gender was conceptualized as potentially relevant to multiple components of the proposed model rather than to a single isolated pathway. Estimating structurally equivalent models separately for boys and girls allowed the overall pattern of associations to be examined within each group without specifying numerous gender interaction and higher-order interaction terms in a single conditional-process model. The establishment of measurement invariance further indicated that the focal constructs were measured comparably across boys and girls, thereby supporting the interpretation of the gender-stratified models within a common measurement framework.

## 3 Results

### 3.1 Preliminary analyses

To examine the potential threat of common method bias, two statistical approaches were employed. First, Harman’s single-factor test was conducted by entering all items from the key study variables into an unrotated exploratory factor analysis. The results revealed 13 factors with eigenvalues greater than 1.00, and the first factor accounted for 21.06% of the total variance, which was below the commonly used threshold of 40%. Second, a bifactor CFA model including an unmeasured latent method factor was specified, in which all items loaded simultaneously on their respective trait factors and on a global method factor. The inclusion of the common method factor did not result in a meaningful improvement in model fit (ΔCFI < 0.01, ΔTLI < 0.01, ΔRMSEA < 0.05; Little, 2024), further indicating that common method bias was unlikely to substantially bias the results.

Multigroup confirmatory factor analysis was conducted to test measurement invariance across gender. Configural, metric, scalar, and strict invariance models were sequentially tested. The results supported configural, metric, scalar, and strict invariance across boys and girls (all ΔCFI ≤ 0.01 and ΔRMSEA ≤ 0.015; Chen, 2007; see Supplementary Table 1), suggesting that the study constructs could be compared meaningfully between boys and girls.

### 3.2 Descriptive statistics and correlations

The descriptive statistics and correlations among the study variables are presented in Table 1. For both boys and girls, adolescents’ depressive symptoms were positively correlated with their best friends’ depressive symptoms and rumination, and negatively correlated with friendship quality and adolescents’ own peer acceptance. In addition, independent-samples t-tests indicated significant mean-level differences between boys and girls across all study variables, although the corresponding effect sizes were small in magnitude, with absolute Cohen’s *d* values ranging from 0.13 to 0.38 (see Table 1). Specifically, girls reported significantly higher levels of depressive symptoms, rumination, and friendship quality than boys, and had best friends with higher levels of depressive symptoms. In contrast, boys had significantly higher levels of adolescents’ own peer acceptance and best friends’ peer acceptance.

**Table 1:** Descriptive statistics and correlations.

Variables	Correlations					
	1	2	3	4	5	6
1 Adolescents’ own Dep	1	0.19^***^	0.72^***^	−0.27^***^	−0.17^***^	−0.14^**^
2 Best friends’ Dep	0.14^**^	1	0.11^*^	−0.10^*^	−0.10^*^	−0.15^**^
3 Rumination	0.61^***^	0.10^*^	1	−0.11^*^	−0.05	−0.05
4 Friendship quality	−0.22^***^	−0.03	−0.11^*^	1	0.18^***^	0.09
5 Adolescents’ own peer Acp	−0.11^**^	−0.11^*^	−0.03	0.18^***^	1	0.28^***^
6 Best friends’ peer Acp	−0.05	−0.15^**^	−0.04	0.04	0.29^***^	1
M ± SD (Boys)	0.65 ± 0.38	0.64 ± 0.37	1.71 ± 0.51	3.50 ± 0.65	0.11 ± 1.02	0.47 ± 0.91
M ± SD (Girls)	0.78 ± 0.42	0.76 ± 0.42	1.87 ± 0.52	3.74 ± 0.61	−0.02 ± 0.93	0.18 ± 0.91
*t*	−4.98	−4.84	−4.86	−6.09	2.01	5.04
*p*	<0.001	<0.001	<0.001	<0.001	0.045	<0.001
Cohen’s *d*	−0.32	−0.30	−0.31	−0.38	0.13	0.32

^***^p < 0.001, ^**^p < 0.01, ^*^p < 0.05. Dep = depressive symptoms; Acp = acceptance. Correlations for girls are shown above, correlations for boys are shown below. Cohen’s d was calculated as the mean for boys minus the mean for girls; negative values indicate higher scores among girls.

### 3.3 Mediating effects of friendship quality and rumination

The hypothesized parallel mediation pathways involving friendship quality and rumination were evaluated using Model 4 of the PROCESS macro. Path estimates are presented in Table 2, and indirect effects are presented in Table 3. For boys, best friends’ depressive symptoms were not indirectly associated with their own depressive symptoms through friendship quality or rumination. For girls, however, significant parallel indirect associations were observed through friendship quality and rumination. The full outcome equations accounted for 52% of the variance in boys’ depressive symptoms and 55% of the variance in girls’ depressive symptoms. These *R*^2^ values reflect the variance jointly accounted for by best friends’ depressive symptoms, friendship quality, rumination, and the covariates included in the respective models.

**Table 2:** Regression results for the mediation model.

Genders	Regression model		*R^2^*	*F*	*b*	SE	95%CI
	Outcomes variable	Predictors					
Boys	Friendship quality (M1)	Best friends’ Dep (X)	0.002	0.35	−0.06	0.080	[−0.22, 0.09]
	Rumination (M2)	Best friends’ Dep	0.03	5.13^***^	0.11	0.062	[−0.01, 0.23]
	Adolescents’ own Dep (Y)	Best friends’ Dep	0.52	102.15^***^	0.08^*^	0.033	[0.01, 0.14]
		Friendship quality			−0.09^***^	0.019	[−0.13, −0.06]
		Rumination			0.52^***^	0.025	[0.47, 0.57]
Girls	Friendship quality	Best friends’ Dep	0.02	2.52^*^	−0.16^*^	0.067	[−0.29, −0.02]
	Rumination	Best friends’ Dep	0.03	5.27^**^	0.13^*^	0.057	[0.02, 0.24]
	Adolescents’ own Dep	Best friend’s Dep			−0.10^**^	0.032	[0.04, 0.16]
		Friendship quality	0.55	112.28^***^	−0.12^**^	0.022	[−0.16, −0.08]
		Rumination			0.56^***^	0.026	[0.51, 0.61]

^***^p < 0.001, ^**^p < 0.01, ^*^p < 0.05. Dep = depressive symptoms, Acp = acceptance. All regression models included grade and only-child status as covariates.

**Table 3:** Bootstrap tests of indirect effects in the mediation model.

Genders	Path	Effect value	BootSE	BootLLCI	BootULCI
Boys	Total effect	0.14	0.05	0.05	0.23
	Direct effect	0.08	0.03	0.01	0.14
	Total indirect effect	0.06	0.04	−0.01	0.14
	X → M1 → Y	0.01	0.01	−0.01	0.02
	X → M2 → Y	0.06	0.04	−0.01	0.13
Girls	Total effect	0.19	0.05	0.10	0.28
	Direct effect	0.10	0.03	0.04	0.16
	Total indirect effect	0.09	0.03	0.03	0.16
	X → M1 → Y	0.02	0.01	0.003	0.04
	X → M2 → Y	0.07	0.03	0.01	0.14

Table 3 presents the indirect effects, standard errors, and 95% CIs. For girls, the indirect effect via friendship quality was 0.02 (*SE* = 0.01, 95% CI [0.003, 0.04]), with the confidence interval excluding zero. The indirect effect via rumination was 0.07 (*SE* = 0.03, 95% CI [0.01, 0.14]), also excluding zero. The proportion of the total association accounted for by the indirect effect was 10.53% for friendship quality and 36.84% for rumination. These findings indicate that significant parallel indirect associations were observed through friendship quality and rumination in the association between best friends’ depressive symptoms and girls’ own depressive symptoms, but not in the corresponding association for boys.

### 3.4 Moderating effect of adolescents’ own peer acceptance and best friends’ peer acceptance

**Figure 2:**
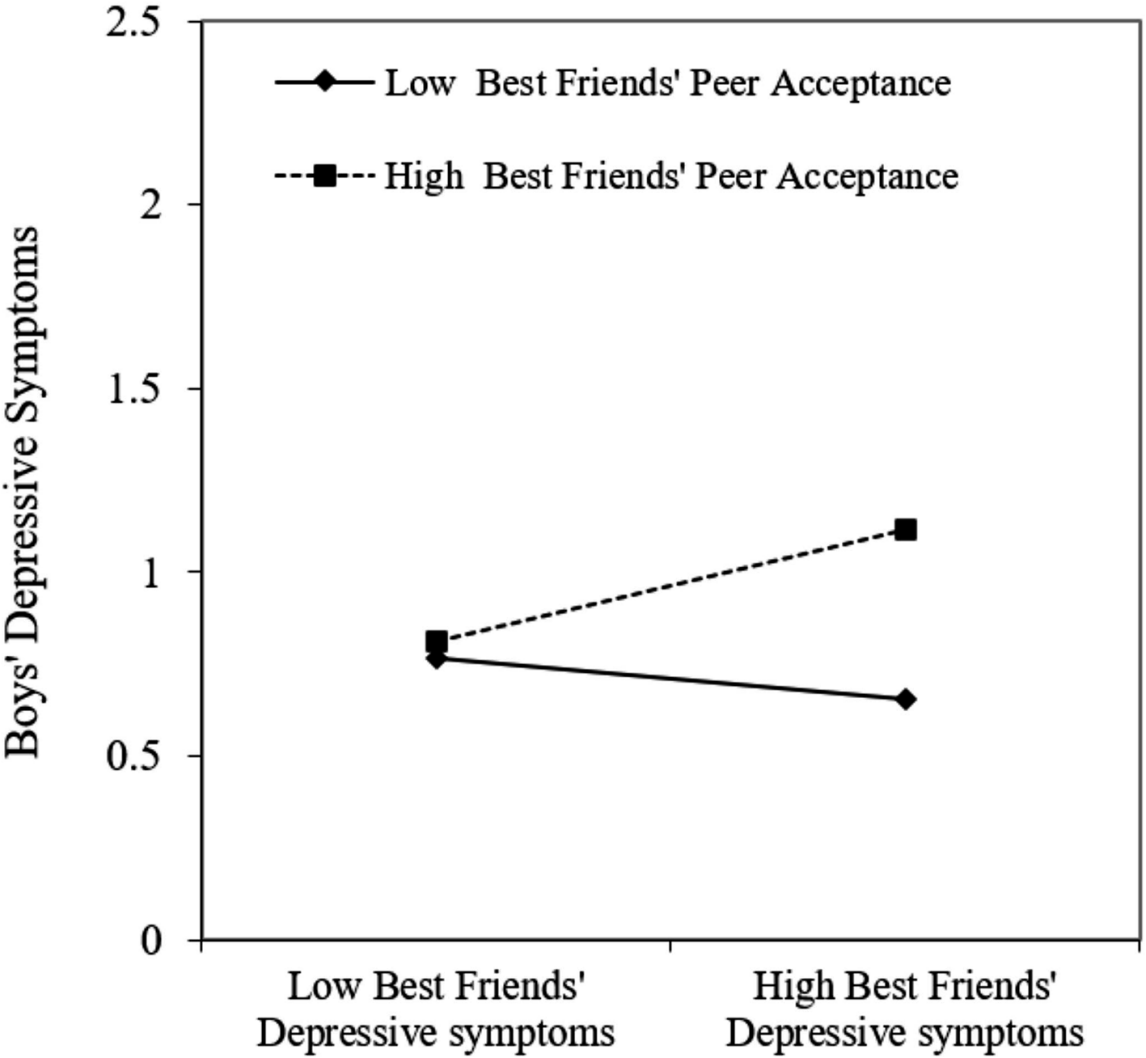
Simple slopes for the interaction between best friends’ depressive symptoms and best friends’ peer acceptance among boys.

A moderated mediation model was tested using Model 8 of the PROCESS macro, with best friends’ peer acceptance as the moderator (see Table 4). For boys, no significant conditional indirect effects were found. However, best friends’ peer acceptance significantly moderated the direct association between best friends’ depressive symptoms and boys’ own depressive symptoms (*b* = 0.03, *SE* = 0.015, *p* < 0.05, 95% CI [0.001, 0.06]). No significant moderating effects were found for the other paths (all *p*-values > 0.05). Simple-slopes analysis (see Figure 2) revealed that the positive association between best friends’ depressive symptoms and boys’ own depressive symptoms was significant when best friends’ peer acceptance was high (+1*SD*; *b* = 0.10, *t* = 3.08, *p* = 0.002), but not when best friends’ peer acceptance was low (−1*SD*; *b* = −0.04, *t* = −1.01, *p* = 0.32). For girls, no significant moderating effect of best friends’ peer acceptance was observed on any path (all *p*-values > 0.05).

**Table 4:** Regression results testing the moderating role of best friends’ peer acceptance.

Genders	Regression model		*R^2^*	*F*	*b*	SE	95%CI
	Outcomes	Predictors					
Boys	Friendship quality (M1)	Best friends’ Dep (X)	0.004	0.34	−0.04	0.053	[−0.15, 0.06]
		Best friends’ peer Acp (W1)			0.04	0.054	[−0.06, 0.15]
		Int1(X × W1)			0.02	0.055	[−0.08, 0.13]
	Rumination (M2)	Best friends’ Dep	0.04	3.69^**^	0.09	0.051	[−0.01, 0.19]
		Best friends’ peer Acp			−0.08	0.051	[−0.18, 0.02]
		Int2(X × W1)			−0.06	0.052	[−0.16, 0.04]
	Adolescents’ own Dep (Y)	Best friends’ Dep	0.52	73.90^***^	0.02	0.014	[−0.01, 0.05]
		Friendship quality			−0.06^***^	0.012	[−0.09, −0.04]
		Rumination			0.27^***^	0.013	[0.25, 0.30]
		Best friends’ peer Acp			0.01	0.014	[−0.02, 0.04]
		Int3(X × W1)			0.03^*^	0.015	[0.001, 0.06]
Girls	Friendship quality	Best friends’ Dep	0.02	2.21^*^	−0.09^*^	0.043	[−0.18, −0.01]
		Best friends’ peer Acp			0.07	0.049	[−0.02, 0.17]
		Int1(X × W1)			0.04	0.048	[−0.05, 0.14]
	Rumination	Best friends’ Dep	0.04	3.54^**^	0.09^*^	0.045	[0.001, 0.18]
		Best friends’ peer Acp			−0.05	0.051	[−0.15, 0.06]
		Int2(X × W1)			0.06	0.050	[−0.04, 0.15]
	Adolescents’ own Dep	Best friends’ Dep	0.56	81.98^***^	0.04^**^	0.013	[0.01, 0.06]
		Friendship Quality			−0.08^***^	0.014	[−0.10, −0.05]
		Rumination			0.29^***^	0.013	[0.26, 0.32]
		Best friends’ peer Acp			−0.04^*^	0.015	[−0.07, −0.01]
		Int3(X × W1)			0.01	0.014	[−0.02, 0.04]

^***^p < 0.001, ^**^p < 0.01, ^*^p < 0.05. Dep = depressive symptoms, Acp = acceptance. All regression models included grade and only-child status as covariates.

**Figure 3:**
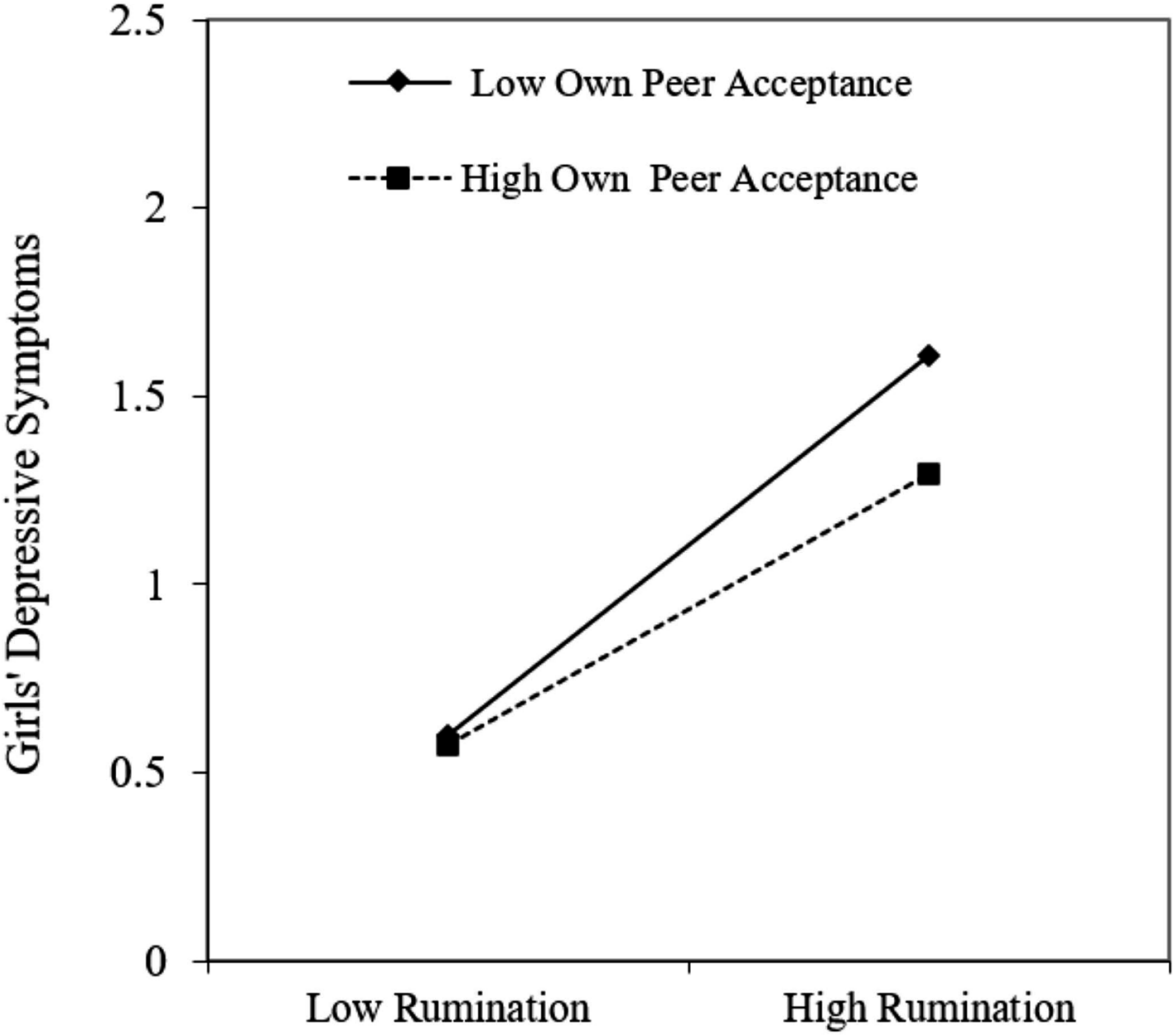
Simple slopes for the interaction between rumination and adolescents’ own peer acceptance among girls.

Another moderated mediation model was tested using Model 15 of the PROCESS macro, with adolescents’ own peer acceptance as the moderator (see Table 5). The results revealed a significant moderating effect only on the association between rumination and girls’ own depressive symptoms (*b* = − 0.03, *SE* = 0.014, *p* < 0.05, 95% CI [− 0.06, − 0.01]). No significant moderating effects were found for the other paths (all *p* values > 0.05). Simple-slopes analysis (see Figure 3) revealed that the positive association between rumination and girls’ own depressive symptoms was weaker for girls with high peer acceptance (+1*SD*; *b* = 0.24, *t* = 9.43, *p* < 0.001) than for those with low peer acceptance (−1*SD*; *b* = 0.34, *t* = 13.22, *p* < 0.001). Specifically, the positive association between rumination and depressive symptoms was weaker at higher levels of peer acceptance.

**Table 5:** Regression results testing the moderating role of adolescents’ own peer acceptance.

Genders	Regression model		*R^2^*	*F*	*b*	SE	95%CI
	Outcomes	Predictors					
Boys	Friendship quality (M1)	Best friends’ Dep (X)	0.002	0.35	−0.04	0.050	[−0.14, 0.06]
	Rumination (M2)	Best friends’ Dep	0.03	5.13^**^	0.08	0.047	[−0.01, 0.17]
	Adolescents’ own Dep (Y)	Best friends’ Dep	0.52	57.23^***^	0.03^*^	0.013	[0.001, 0.05]
		Friendship quality			−0.06^***^	0.013	[−0.08, −0.03]
		Rumination			0.27^***^	0.013	[0.24, 0.29]
		Adolescents’ own peer Acp (W2)			−0.01	0.013	[−0.04, 0.01]
		Int1(X × W2)			0.02	0.013	[−0.009, 0.04]
		Int2(M1 × W2)			0.001	0.013	[−0.02, 0.03]
		Int3(M2 × W2)			−0.003	0.012	[−0.03, 0.02]
Girls	Friendship quality	Best friends’ Dep	0.02	2.52^*^	−0.10^*^	0.042	[−0.18, −0.02]
	Rumination	Best friends’ Dep	0.03	5.27^**^	0.10^*^	0.044	[0.01, 0.18]
	Adolescents’ own Dep	Best friends’ Dep	0.57	66.00^***^	0.04^**^	0.013	[0.02, 0.07]
		Friendship quality			−0.07^***^	0.014	[−0.10, −0.04]
		Rumination			0.29^***^	0.013	[0.26, 0.31]
		Adolescents’ own peer Acp			−0.04^**^	0.015	[−0.07, −0.01]
		Int1(X × W2)			0.02	0.014	[−0.01, 0.05]
		Int2(M1 × W2)			0.001	0.015	[−0.03, 0.03]
		Int3(M2 × W2)			−0.03^*^	0.014	[−0.06, −0.01]

^***^p < 0.001, ^**^p < 0.01, ^*^p < 0.05. Dep = depressive symptoms, Acp = acceptance. All regression models included grade and only-child status as covariates.

The indirect effect of best friends’ depressive symptoms on girls’ own depressive symptoms through rumination was significant at low (indirect effect = 0.031, *SE* = 0.014, 95% CI [0.01, 0.06]), mean (indirect effect = 0.029, *SE* = 0.013, 95% CI [0.005, 0.05]), and high levels of peer acceptance (indirect effect = 0.025, SE = 0.011, 95% CI [0.004, 0.05]), as none of the confidence intervals contained zero. Importantly, the magnitude of this indirect effect decreased as peer acceptance increased, indicating that the conditional indirect association through rumination was smaller at higher levels of peer acceptance. Specifically, the estimated indirect association was largest at low peer acceptance and smallest at high peer acceptance.

## 4 Discussion

To extend and refine understanding of how depressive symptoms are linked within adolescent best friendships, the present study examined the association between best friends’ depressive symptoms and adolescents’ own depressive symptoms in relation to dyadic friendship processes and broader peer-status contexts. The findings revealed different patterns across the gender-stratified analyses. Among girls, best friends’ depressive symptoms were indirectly associated with girls’ own depressive symptoms through poorer friendship quality and greater rumination. Among boys, these indirect associations were not significant; instead, the direct association between best friends’ depressive symptoms and boys’ depressive symptoms was stronger when best friends’ peer acceptance was higher. Adolescents’ own peer acceptance showed a different pattern among girls: the association between rumination and depressive symptoms and the corresponding indirect association were weaker at higher levels of peer acceptance.

### 4.1 Gender-stratified patterns in the roles of friendship quality and rumination

The present findings point to distinct interpersonal and cognitive patterns within the gender-stratified analyses. Among girls, best friends’ depressive symptoms were indirectly associated with girls’ own depressive symptoms through both friendship quality and rumination. The indirect association through friendship quality was consistent with interpersonal theories of depression, which propose that depressive symptoms may place strain on close relationships through negative interpersonal exchanges (Coyne, 1976), and with evidence linking friendship experiences to youths’ depressive symptoms (Li et al., 2025; Schwartz-Mette et al., 2020). This pathway may be especially relevant for girls, as their friendships tend to involve greater intimacy, self-disclosure, emotional support, and problem-focused talk (Buhrmester, 1990; Rose et al., 2016; Rose and Rudolph, 2006). Within such emotionally engaged friendships, best friends’ depressive symptoms may undermine perceived friendship quality, which in turn may be associated with higher depressive symptoms among girls.

The significant indirect effect through rumination further supports a cognitive pathway that may be particularly relevant for girls. This pattern among girls is consistent with prior evidence that girls tend to report higher levels of rumination than boys during adolescence (Jose and Brown, 2008; Rood et al., 2009), and with meta-analytic evidence that girls generally show greater ruminative tendencies than boys, which may help explain their elevated risk for depressive symptoms (Johnson and Whisman, 2013). More specifically, brooding rumination has been identified as a mechanism linking stress exposure to later internalizing symptoms (Fredrick et al., 2024), suggesting that repeatedly focusing on negative affect may link interpersonal stress with depressive vulnerability. In the context of close friendships, emotionally intensive discussions such as co-rumination may further reinforce such ruminative processing and amplify internalizing symptoms, a process likely to be especially salient for girls given their greater tendency to co-ruminate (Bastin et al., 2021; Schwartz-Mette and Rose, 2012). Thus, girls exposed to best friends’ depressive symptoms may be especially likely to process such emotional content through ruminative thinking, which in turn is associated with their own depressive symptoms.

For boys, no significant indirect association emerged through either friendship quality or rumination. This finding does not suggest that interpersonal or cognitive vulnerabilities are unimportant for boys, as both friendship quality and rumination were associated with boys’ depressive symptoms. Rather, these factors may not be the primary pathways through which best friends’ depressive symptoms are associated with boys’ own symptoms. Prior research suggests that boys may be less likely to engage in emotionally intensive problem talk or to process a friend’s distress through repeated discussion and rumination (Rose et al., 2016; Rose and Rudolph, 2006). Associations with a depressed friend may therefore operate through other processes not captured in the present model, such as withdrawal from shared activities, reduced companionship, or broader peer-group dynamics. Future research should examine these alternative pathways and consider whether smaller indirect effects among boys require greater statistical power to detect.

Because the corresponding coefficients were not formally compared across gender, differences in statistical significance between the gender-stratified models should not be interpreted as evidence of statistically significant cross-gender differences.

### 4.2 Best friends’ peer acceptance as a contextual moderator among boys

A noteworthy finding was that best friends’ peer acceptance moderated the association between best friends’ depressive symptoms and boys’ own depressive symptoms. Specifically, best friends’ depressive symptoms were positively associated with boys’ depressive symptoms only when best friends were highly accepted by their peers. This pattern suggests that a best friend’s broader peer standing may shape the extent to which boys are susceptible to their best friend’s depressive symptoms.

One possible explanation is that highly accepted friends are more socially salient and valued within the peer group. This finding was consistent with our hypothesis that the association between best friends’ depressive symptoms and adolescents’ own depressive symptoms would be stronger at higher levels of best friends’ peer acceptance and a significant interaction was observed in the boys’ subsample. The emotional expressions and behaviors of highly accepted friends may attract greater attention or carry greater interpersonal weight. This interpretation is consistent with prior longitudinal evidence showing that boys were more susceptible to depressive symptom contagion when their friends were higher in peer-perceived popularity (Prinstein, 2007). Experimental research has similarly shown that adolescent boys display greater conformity and internalization when attitudes are endorsed by high-status rather than low-status peers (Cohen and Prinstein, 2006). Although peer acceptance is conceptually distinct from perceived popularity or status, these constructs all capture aspects of adolescents’ broader social position within the peer group (Cillessen and Rose, 2005). Thus, a highly accepted best friend may be relevant not only as a close dyadic partner but also because of their visibility and validation within the broader peer group. Future studies incorporating social network measures of acceptance, popularity, and centrality are needed to clarify whether this moderating pattern is related to greater visibility, social embeddedness, or access to shared peer contexts.

### 4.3 Adolescents’ own peer acceptance as a protective context among girls

Girls’ own peer acceptance appeared to function as a pathway-specific protective context. Peer acceptance did not broadly buffer all peer-related pathways; rather, the association between rumination and depressive symptoms was weaker at higher levels of peer acceptance, and the corresponding conditional indirect association between best friends’ depressive symptoms and girls’ depressive symptoms through rumination was smaller. This pattern is consistent with empirical evidence indicating that rumination is more strongly implicated in depressive vulnerability among adolescent girls than boys (Jose and Brown, 2008).

One explanation is that peer acceptance may reduce the salience of the social threats on which rumination often focuses. Rumination often involves repetitive attention to negative emotions, interpersonal problems, and possible rejection. For girls with low peer acceptance, such repetitive thinking may more easily reinforce feelings of exclusion, insecurity, or low social worth. In contrast, girls who are accepted by the broader peer group may have stronger social validation and belonging, which may help explain why rumination was less strongly associated with depressive symptoms. This interpretation is supported by longitudinal evidence that peer rejection predicts later depressive symptoms among adolescents, particularly when paired with cognitive vulnerability (Prinstein and Aikins, 2004), and by research showing that adolescent girls’ depressive symptoms are closely tied to interpersonal vulnerabilities and peer relationship experiences (Prinstein et al., 2005). In addition, recent evidence indicates that peer acceptance makes a unique contribution to lower depressive symptoms and that overall peer relationship quality shows a stronger protective association for girls than for boys (Adedeji et al., 2022). Thus, the potentially protective role of peer acceptance may lie not in lower rumination itself, but in a weaker association between rumination and depressive symptoms.

These findings refine the understanding of depression-related peer influence by suggesting that peer acceptance may not operate as a uniform marker of risk or protection, but carries different implications depending on whether it refers to best friends or adolescents themselves. Best friends’ peer acceptance may increase the social salience and interpersonal weight of their depressive symptoms, as suggested by the pattern observed in the boys’ subsample, for whom friends’ broader peer status and visibility may be especially influential (Cillessen and Rose, 2005; Prinstein, 2007). In contrast, girls’ own peer acceptance may provide social validation, belonging, and support, which may help explain the weaker association between rumination and depressive symptoms at higher levels of peer acceptance. This interpretation is consistent with evidence that girls place greater value on intimacy and support in peer relationships (Rose and Rudolph, 2006). Extending prior work showing that depressive symptoms and depressogenic styles can become shared within adolescent friendships (e.g., Giletta et al., 2011; Stevens and Prinstein, 2005), the present findings suggest that such associations should be understood as status-contingent patterns of association embedded in both dyadic friendships and the broader peer group.

### 4.4 Strengths, implications, limitations, and future directions

The present study has several strengths. First, it examined potential interpersonal and cognitive pathways involved in the association between best friends’ depressive symptoms and adolescents’ own depressive symptoms by distinguishing between friendship quality and rumination. Second, the study extended the analysis beyond the best-friend dyad by incorporating peer acceptance as an indicator of broader peer-group status. Importantly, it considered both best friends’ peer acceptance and adolescents’ own peer acceptance, allowing the study to distinguish the social influence associated with friends’ status from the protection associated with adolescents’ own social position. Third, the study revealed different patterns across the gender-stratified analyses, suggesting that depression-related associations within friendships may take different forms within the boys’ and girls’ subsamples. Together, these strengths suggest that depression-related peer associations in adolescence require attention to close friendship processes, broader peer-group status, and possible gender-related variation.

The findings suggest the value of a more relational and context-sensitive approach to school-based prevention. Rather than viewing adolescents’ depressive symptoms solely as an individual concern, prevention programs may also consider how emotional vulnerability is associated with close friendship functioning and the broader peer context in which friendships are embedded. School-based programs may incorporate cognitive strategies that help adolescents recognize and manage repetitive negative thinking, together with support for healthier friendship functioning, such as constructive communication, adaptive responses to interpersonal distress, and access to multiple sources of social support. The findings involving adolescents’ own and their best friends’ peer acceptance further suggest that practitioners may benefit from considering not only the quality of close friendships but also the broader peer-group contexts surrounding both friendship partners. Taken together, these findings may inform a multilevel approach to school-based prevention that integrates individual cognitive support, friendship-sensitive intervention, and attention to the wider peer context.

Although several associations were statistically significant, the indirect associations via friendship quality and rumination were modest in magnitude and should be interpreted as small components of a broader multifactorial process rather than as strong or deterministic effects. The full outcome equations accounted for 52 and 55% of the variance in boys’ and girls’ depressive symptoms, respectively; however, these values reflect the joint contribution of all predictors in the models and should not be attributed specifically to the indirect pathways. Given that adolescent depressive symptoms are associated with multiple individual, relational, familial, and contextual factors, the present findings support considering friendship quality and rumination as components of broader, multilevel prevention frameworks rather than as isolated intervention targets.

Despite these strengths, several limitations merit acknowledgment. First, the cross-sectional design precludes conclusions about directionality or causality and cannot distinguish peer influence from peer selection, reciprocal effects, or shared contextual factors. Accordingly, the estimated indirect effects should be interpreted as cross-sectional statistical associations consistent with the hypothesized mediation model, rather than as evidence of temporal or causal mediation. Future longitudinal, cross-lagged, or social network studies are needed to clarify how depressive symptoms and related processes unfold across friendships over time. Second, although this study examined rumination as an individual cognitive-emotional process, it did not assess co-rumination, a dyadic conversational pattern that may also link friends’ depressive symptoms (Rose, 2002; Stone et al., 2011). Future research should examine rumination and co-rumination together. Third, the study identified only one classroom best friend for each adolescent. Although this approach captures a salient dyadic relationship, it simplifies adolescents’ broader friendship networks and may overlook the concurrent influence of other close friends, friendship groups, and the wider peer network (Conway et al., 2011; Kiuru et al., 2012). Future research should combine multiple-friend nominations with social network measures to disentangle the contribution of a nominated best friend from that of the broader peer context. Fourth, the use of unilateral best-friend nominations precluded distinguishing reciprocated from non-reciprocated friendships. This distinction may be consequential because reciprocal and unilateral ties can differ in intimacy, support, and perceived relational investment, which may condition the strength of interpersonal associations (Vaquera and Kao, 2008; Meter et al., 2015). Future studies should examine friendship reciprocity as a relational moderator and model associations separately across reciprocal and unilateral ties. Fifth, although the proposed models were estimated separately for boys and girls, the corresponding path coefficients were not formally compared across gender. Therefore, differences in the statistical significance or magnitude of estimates across the gender-stratified models should not be interpreted as evidence of statistically significant between-gender differences. Future research could use formal multigroup comparisons to determine whether the relevant direct, indirect, and conditional associations differ significantly between boys and girls.

## 5 Conclusion

The present study contributes to the literature by situating best-friend depressive symptom similarity within both dyadic friendship processes and broader peer-status contexts. Rather than suggesting a uniform association between best friends’ and adolescents’ depressive symptoms, the findings revealed different patterns across the gender-stratified analyses and according to whose peer acceptance was considered. For girls, the link with best friends’ depressive symptoms was embedded in both poorer friendship quality and greater rumination, whereas girls’ own peer acceptance appeared to reduce the depressive significance of rumination. For boys, the role of best friends’ peer acceptance points to the importance of considering the social position of influential peers within the larger peer group. Together, these findings suggest that adolescent depressive symptoms should be understood in relation to both close friendship processes and adolescents’ positions within the broader peer group. Prevention efforts may benefit from considering the quality of close friendships as well as the peer acceptance of both adolescents and their best friends.

## Data Availability

The original contributions presented in the study are included in the article/Supplementary material, further inquiries can be directed to the corresponding author.
