# Supplementary material for: Best friends’ and adolescents’ depressive symptoms in early adolescence: gendered pathways and distinct roles of peer acceptance
Source: Front Psychol. 2026 Aug 19;17:1909249. doi: 10.3389/fpsyg.2026.1909249 (PMC13533713; doi:10.3389/fpsyg.2026.1909249)
Supplement: Supplementary file 1 [file Table_1.DOCX]

Supplementary Material

# 1 Supplementary Table

**Supplementary Table 1.** Model Fit Comparisons for Measurement Invariance Across Gender

| Models | χ^2^ | *df* | *p* | RMSEA | CFI | TLI | ΔCFI | ΔRMSEA |
| --- | --- | --- | --- | --- | --- | --- | --- | --- |
| **Depressive symptoms** |  |  |  |  |  |  |  |  |
| M1: Configural Invariance | 490.57 | 328 | < 0.001 | 0.031 | 0.95 | 0.94 |  |  |
| M2: Weak Invariance | 520.67 | 348 | < 0.001 | 0.031 | 0.95 | 0.94 | -0.003 | 0.000 |
| M3: Strong Invariance | 578.65 | 368 | < 0.001 | 0.033 | 0.94 | 0.94 | -0.006 | 0.002 |
| M4: Strict Invariance | 630.70 | 388 | < 0.001 | 0.035 | 0.93 | 0.93 | -0.009 | 0.002 |
| **Rumination** |  |  |  |  |  |  |  |  |
| M1: Configural Invariance | 855.53 | 408 | < 0.001 | 0.046 | 0.92 | 0.91 |  |  |
| M2: Weak Invariance | 876.53 | 430 | < 0.001 | 0.045 | 0.92 | 0.92 | 0.000 | -0.001 |
| M3: Strong Invariance | 931.77 | 452 | < 0.001 | 0.046 | 0.92 | 0.91 | -0.006 | 0.001 |
| M4: Strict Invariance | 963.37 | 474 | < 0.001 | 0.045 | 0.91 | 0.92 | -0.002 | -0.001 |
| **Friendship quality** |  |  |  |  |  |  |  |  |
| M1: Configural Invariance | 513.61 | 236 | < 0.001 | 0.048 | 0.93 | 0.90 |  |  |
| M2: Weak Invariance | 522.93 | 254 | < 0.001 | 0.046 | 0.93 | 0.91 | 0.002 | -0.002 |
| M3: Strong Invariance | 616.70 | 270 | < 0.001 | 0.050 | 0.91 | 0.90 | -0.018 | 0.004 |
| M4: Strict Invariance | 668.50 | 286 | < 0.001 | 0.051 | 0.90 | 0.90 | -0.01 | 0.001 |
